# Sexual dysfunction in movement disorders: prevalence, clinical features, associated factors and management

**DOI:** 10.1016/j.prdoa.2026.100452

**Published:** 2026-05-09

**Authors:** Vincent Leclercq

**Affiliations:** a Department of Neurology, Hôpital Universitaire de Bruxelles - Hôpital Erasme, Brussels, Belgium; b Laboratory of Experimental Neurology, Université Libre de Bruxelles (ULB), Brussels, Belgium

**Keywords:** Parkinson’sdisease, Movementdisorders, Sexualdysfunction, Hypersexuality, Non-motorsymptoms, Dopamineagonists, Impulsecontroldisorders, Sexualhealth

## Abstract

Sexual dysfunction is a common but insufficiently addressed non-motor symptom in movement disorders. While Parkinson’s disease (PD) is the condition most frequently investigated, sexual dysfunction is also reported in Huntington’s disease, dystonia, and other movement disorders. Clinical presentations are heterogeneous, encompassing reduced desire, erectile dysfunction, ejaculatory and orgasmic disorders, lubrication difficulties, dyspareunia, and hypersexuality. In PD, prevalence estimates vary widely across studies, largely because of differences in disease stage, cultural context, and assessment methodology. The most consistent associated factors include depression, anxiety, age, autonomic dysfunction, and motor disability. Dopaminergic treatment has a bidirectional influence, with potential benefit on hypoactive sexual symptoms but an increased risk of hypersexuality, especially with dopamine agonists. Evidence for treatment remains limited, with the strongest support for phosphodiesterase-5 inhibitors in erectile dysfunction and dopaminergic treatment adjustment in hypersexuality. Sexual dysfunction remains underrecognized in routine practice despite its major impact on quality of life and intimate relationships. Routine screening and multidisciplinary management should be encouraged in the care of patients with movement disorders.

## Highlights

• Sexual dysfunction is a frequent but underrecognized non-motor symptom across movement disorders, particularly in Parkinson’s disease.
• In Parkinson’s disease, reported prevalence ranges widely from 23% to 95%, reflecting major methodological and population heterogeneity.
• Clinical manifestations include both hypoactive symptoms (reduced desire, erectile dysfunction, arousal and orgasmic difficulties) and hypersexuality.
• Depression, anxiety, age, autonomic dysfunction and motor severity are the most consistent factors associated with sexual dysfunction.
• Available treatment data remain limited, highlighting the need for routine screening and multidisciplinary management in clinical practice.

## 1 Introduction

**Fig. 1:**
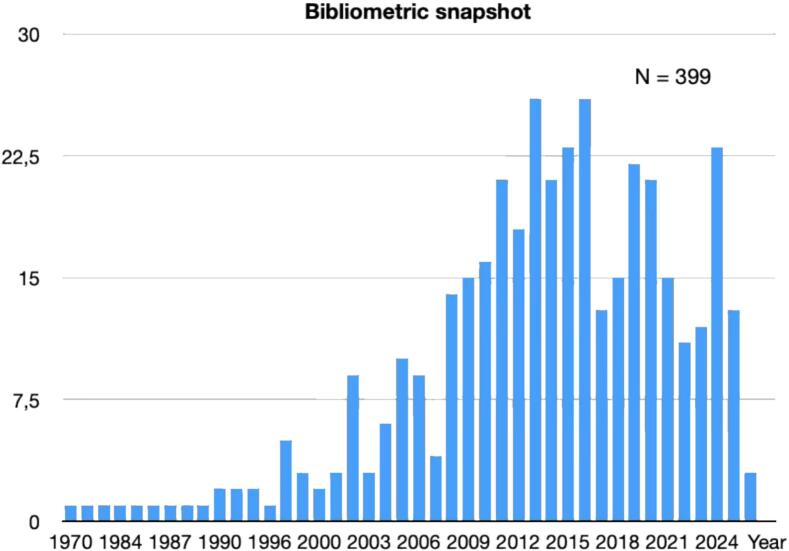
Bibliometric snapshot. Counts represent PubMed-indexed records retrieved on April 19, 2026 using the following query strings “(“Parkinson*” OR “Multiple system atrophy” OR “Progressive supranuclear palsy” OR “Essential tremor” OR “Huntington” OR “Dystonia” OR “Movement disorder”) AND (“sexual satisfaction” OR “Erectile dysfunction” OR “female sexual dysfunction” OR “libido” OR “orgasm*” OR “hypersexual*” OR “Impulse control”)”.

Sexual health is a fundamental component of quality of life, well-being, intimate relationships, and personal identity [1], [2]. Yet in neurology, and particularly in the field of movement disorders, sexual dysfunction remains insufficiently explored in both research and clinical practice [2], [3], [4], [5], [6]. While motor manifestations often dominate diagnostic and therapeutic discussions, patients with movement disorders frequently experience a broad range of non-motor symptoms that may be equally disabling. Among these, sexual dysfunction is especially important because it affects self-esteem, emotional well-being, couple dynamics, and overall life satisfaction, while often remaining unreported and untreated [3], [7], [8], [9]. Fig. 1..

Movement disorders may interfere with sexual function through multiple and often overlapping mechanisms. Neurodegenerative and network-level changes can directly affect desire, arousal, orgasm, and sexual behavior, while autonomic dysfunction, motor disability, pain, fatigue, sleep disturbance, psychiatric comorbidity, endocrine changes, and medication effects may further contribute. In addition, sexual symptoms in this population are not limited to hypoactive disorders such as reduced libido, erectile dysfunction (ED), lubrication problems, or anorgasmia [1], [10]. In some patients, particularly those exposed to dopaminergic therapies, pathological increases in sexual drive or behavior may emerge, including hypersexuality as part of the spectrum of impulse control disorders (ICDs) [1], [2], [8], [10]. Sexual dysfunction in movement disorders should therefore not be viewed as a single entity, but rather as a heterogeneous and multidimensional clinical domain.

Parkinson’s disease (PD) is the condition in which sexual dysfunction has been most extensively studied, with reports describing a high prevalence of both hypoactive and hyperactive sexual symptoms in men and women [10], [11], [12], [13], [14]. However, sexual dysfunction is not confined to PD. Available evidence also suggests a substantial burden in Huntington’s disease, dystonia, and other movement disorders, although these conditions remain far less investigated [15], [16], [17], [18].

The reported rates of sexual dysfunction differ significantly across studies, mainly due to diverse populations and measurement methods [2], [7], [9], [19]. Additionally, dopaminergic treatments can have dual effects, possibly alleviating hypoactive symptoms but also raising the risk of hypersexuality or similar impulse control issues in susceptible individuals [11], [12], [20], [21]. In this narrative review, we outline the epidemiology, clinical characteristics, underlying mechanisms, associated factors and practical management approaches, focusing on feasible screening and referral processes.

## 2 Methods

A narrative review based on a targeted PubMed literature search conducted in April 2026 was performed, supplemented by backward citation tracking of key reviews and original articles. Search items combined movement disorder diagnoses (“Parkinson*”, “multiple system atrophy”, “progressive supranuclear palsy”, “essential tremor”, “Huntington*”, “Dystonia”, “Movement disorder”) with sexual health keywords (“sexual satisfaction”, “erectile dysfunction”, “female sexual dysfunction”, “libido”, “orgasm*”, “hypersexual*”, “impulse control”). Peer-reviewed human studies reporting prevalence, clinical correlates, pathophysiology or management of sexual dysfunction were prioritized, including observational studies, clinical trials, systematic reviews and *meta*-analyses. Selected case reports and case series were retained when they addressed uncommon but clinically relevant phenomena, particularly hypersexuality. Because the aim was to provide a clinically oriented narrative synthesis, no formal risk-of-bias assessment or PRISMA-style study flow was performed. The final synthesis included 194 cited sources.

## 3 Pathophysiology

Sexual function depends on coordinated integration of motivational processing, autonomic regulation, sensory feedback, endocrine status and motor capacity, mediated through a complex interplay of cortical, subcortical, spinal and peripheral neural structures [1], [2], [10]. Across movement disorders, sexual dysfunction arises from multiple interconnected pathophysiological mechanisms.

First, neurodegenerative involvement of limbic-striatal circuits and related neurotransmitter systems modulates desire and reward sensitivity. In PD, functional neuroimaging demonstrates reduced connectivity between dopaminergically innervated regions including the putamen, hypothalamus, nucleus accumbens and frontal cortex in patients with sexual dysfunction, with hypothalamic dysfunction particularly affecting libido and erectile function through altered dopamine-oxytocin pathways [22], [23]. Beyond dopamine, serotonergic and noradrenergic systems play critical modulatory roles. Serotonergic degeneration within corticostriatal limbic areas correlates with severity of apathy, depression and anxiety in PD, and serotonin primarily exerts inhibitory effects on sexual arousal and orgasm through 5-HT1A and 5-HT2A receptors, while norepinephrine contributes to both central arousal and peripheral genital responses [2], [24], [25].

Second, autonomic dysfunction affects genital engorgement, lubrication and orgasmic physiology through disruption of sympathetic (thoracolumbar T11-L2) and parasympathetic (sacral S2-S4) pathways that mediate vascular smooth muscle relaxation and secretomotor responses. The severity of sexual dysfunction correlates significantly with overall autonomic impairment across cardiovascular, urinary and gastrointestinal domains (p < 0.001) [2], [23], [26]. Spinal cord mechanisms are essential, with psychogenic arousal mediated by sympathetic preganglionic neurons in the thoracolumbar intermediolateral cell column and dorsal commissural nucleus, reflexogenic arousal mediated by sacral parasympathetic preganglionic neurons and ejaculation controlled by a spinal pattern generator at L3-L5 receiving projections from brainstem and hypothalamus [2], [27]. Autonomic impairment is particularly salient in synucleinopathies with prominent dysautonomia. In multiple system atrophy (MSA), severe autonomic failure including urogenital dysfunction is a core diagnostic criterion reflecting α-synuclein deposition in oligodendrocytes affecting the hypothalamus, thoracolumbar intermediolateral column, and Onuf's nucleus, producing early and extensive central autonomic network degeneration distinct from the predominantly peripheral autonomic involvement in Lewy body disorders [28], [29], [30], [31].

Third, motor disability, fatigue, pain, spasticity and non-motor symptoms including depression, cognitive impairment and lower urinary tract dysfunction, reduce sexual activity and satisfaction through both physical limitations and psychological burden. Disease severity, disease stage, age and depression independently predict sexual dysfunction severity [2], [8], [26].

Fourth, vascular and endocrine mechanisms contribute substantially. Vasculogenic factors include atherosclerotic endothelial dysfunction affecting penile circulation, while endocrinologic factors include testosterone deficiency, hyperprolactinemia and disruption of the hypothalamic-pituitary–gonadal axis, though data on hormonal pathophysiology in PD remain heterogeneous [1], [23], [32], [33].

Fifth, neuroinflammation may contribute to sexual dysfunction pathogenesis. Chronic microglial activation and elevated pro-inflammatory cytokines (TNF-α, IL-1β, IL-6) in PD promote oxidative stress, blood–brain barrier disruption and hypothalamic–pituitary–adrenal axis dysregulation, potentially affecting neuroendocrine control of sexual function, though direct evidence linking neuroinflammation to sexual dysfunction specifically remains limited [34], [35], [36], [37].

Pharmacologic factors further modulate this biology. Dopaminergic treatments may improve hypoactive symptoms in some individuals while increasing risk of hypersexuality and other ICDs in susceptible patients, particularly with dopamine agonists showing D3 receptor selectivity, with cumulative 5-year ICD incidence of 46%, and other medications including SSRIs, antiepileptics, antihypertensives and opiates can impair sexual function through serotonergic modulation, hormonal suppression or vascular effects [2], [12], [13], [24], [38], [39]. Deep brain stimulation of the subthalamic nucleus (STN-DBS) produces modest improvements in sexual function, likely through enhancement of motor function, reduction in dopaminergic medication requirements and improvement in mood and quality of life, though effects are variable and age-dependent [40], [41], [42], [43], [44], [45].

Genetic factors may modulate individual susceptibility to sexual dysfunction in movement disorders. GBA variants (particularly *L444P* mutation) are associated with more frequent sexual dysfunction compared to idiopathic PD or LRRK2-associated PD and polymorphisms in dopamine receptor genes (*DRD1*, *DRD2/ANKK1*, *DRD3*) and glutamate receptor genes (*GRIN2B*) confer increased risk of ICDs including hypersexuality, with genetic models improving predictability of these behaviors when combined with clinical variables [46], [47], [48], [49].

These mechanisms reinforce the need to interpret sexual symptoms as the net result of disease biology, comorbidities, secondary physical factors, psychosocial factors and treatment exposure.

## 4 Prevalence and types of sexual dysfunction

### 4.1 Parkinson’s disease

Sexual dysfunction (SD) is highly prevalent in PD, though reported rates vary substantially depending on assessment tools, sample demographics and study design. Table 1 summarizes prevalence figures from key studies.

**Table 1:** Summary of prevalence from key studies. BDI: Beck Depression Inventory; BISF-M/W: Brief Index of Sexual Functioning for Male/Women; BSFI-M: Brief Male Sexual Function Inventory; ED: erectile dysfunction; EOPD: early-onset Parkinson’s disease; F-SQ: Female Sexual Quotient; FSFI: Female Sexual Function Index; ICD-10: International Statistical Classification of Diseases and Related Health Problems 10th Revision; IIEF: International Index of Erectile Function; MOS-SFS: Medical Outcomes Study Sexual Functioning Scale; OR: odds ratio; PD: Parkinson’s disease; RR: relative risk; SCOPA-AUT: Scales for Outcomes in Parkinson’s Disease – Autonomic Dysfunction; SD: sexual dysfunction.

**Sample (N, setting)**	**Instrument and cutoff scores**	**Prevalence finding**	**Source**
N = 203 (113males,90females), Italy	IIEF ≤ 25 FSFI ≤ 26.55	SD in 68% of men, 53% of women	L. Raciti et al., 2020 [3]
N = 67 cases vs 123 controls, Egypt	ASEX ≥ 19	SD in 64% of patients, 30% of controls	A. M. Elshamy et al., 2021 [50]
N = 40 males, Egypt	IIEF ≤ 25	ED in 70% of men	A. Shalash et al., 2020 [51]
N = 105 EOPD vs 90 controls, Spain	BSFI-M < 3.42 FSFI < 26.5	SD in 70.2% of patients, 52.5% of controls	L. Vela-Desojo et al., 2020 [52]
N = 861 (433males,418females), International	MOS-SFS (at least one complaint)	SD in 52.1% of patients (43.1% in females, 60.7% in males)	T. Kinateder et al., 2022 [53]
N = 411, China	ICD-10 diagnostic criteria	SD in 35.3% of patients	Jun Zhu et al., 2016 [54]
N = 60 cases vs 60 controls, Thailand	ASEX-Thai ≥ 16	SD in 81.6% of patients, 48.3% of controls	O. Jitkritsadakul et al., 2015 [55]
N = 114, Turkey	ASEX (present/absent)	SD in 87–95% of patients	Y. Kaya et al., 2017 [56]
N = 76 (50males,26females), Turkey	ASEX-Turkish > 11	SD in 88.2% of patients	D. Haktanir et al., 2022 [57]
N = 76 (41males,35females), Turkey	ASEX ≥ 19	SD in 53.3% of patients	H. Varlıbaş et al., 2025 [26]
N = 42 (18males,24females), Tunisia	Sexual items of the SCOPA-AUT	SD in 26.2% of patients	A. A. Mousli et al., 2023 [58]
N = 100 (59males,41females), Belgium	ASEX ≥ 19	SD in 23% of patients	R. Van Overmeire et al., 2022 [59]
N = 90 (54males,36females), Brazil	“Loss of libido” from BDI and questions about erectile dysfunction	Loss of libido in 65.6%, ED in 42.6% of men	A. Kummer et al., 2009 [60]
N = 48 cases vs 32 controls, USA	Questions about erectile dysfunction	ED in 60.4% of men, 37.5% in controls	C. Singer et al., 1989 [61]
N = 101 (53males,48females), Poland	Questions about erectile dysfunction	ED in ∼ 85% of men	M. Gołąb-Janowska et al., 2011 [62]
N = 140 males, China	ICD-10 diagnostic criteria	ED in 41.43% of men	X. Hu et al., 2013 [63]
N = 28 female cases vs 28 controls, Brazil	FSFI < 26.5 F-SQ < 60	SD in 60.7% of women, 17.9% of controls	B.S. Panichelli et al., 2026 [64]
N = 121 cases vs 123 controls, Italy	BISF-M > 33.6 BISF-W > 33.6 IIEF ≤ 25 FSFI ≤ 26.55	No significant difference (PD vs. controls)	R. Ferrucci et al., 2016 [65]
Meta-analysis, 22 studies	Various	Pooled OR = 3.5 (PD vs. general population)	M. Vafaeimastanabad et al., 2023 [7]
Meta-analysis, 11 studies	Various	RR = 1.79 in males, not significant in females	S. Zhao et al., 2019 [19]

Prevalence rates range from approximately 23% to 95%, driven primarily by differences in assessment thresholds, cultural context and patient selection. A *meta*-analysis of 22 observational studies calculated a pooled odds ratio of 3.5 (95% CI: 2.19–5.58) for the association between PD and sexual dysfunction compared with the general population [7]. A separate *meta*-analysis found a significant relative risk of 1.79 (95% CI: 1.26–2.54) for SD in males with PD, but no significant association in females (RR = 1.3, 95% CI: 0.64–2.61) [19]. One notable outlier, a four-center Italian study using multiple validated instruments, found no significant differences in total sexual function scores between PD patients and non-Parkinsonian controls, though male patients did show significantly lower orgasm/pleasure domain scores [65]. This study recruited patients with mild-to-moderate PD, which may partly explain the discrepancy with studies enrolling more advanced patients.

The most commonly reported dysfunctions in men include ED (prevalence ranging from 41% to 85%) [51], [61], [62], [63], premature ejaculation (40.6–71%) [66], [67], [68], decreased libido (65.6–84%) [60], [69], and orgasmic dysfunction (39.5–87%) [66], [69]. In men with young-onset PD, 46.8% had ED and 71% had premature ejaculation by Premature Ejaculation Diagnostic Tool (PEDT) criteria [67]. In women, arousal difficulties (up to 87.5%), orgasmic dysfunction (up to 75%), decreased desire (36.8–52.7%), lubrication problems and pain during intercourse are prominent [52], [66], [70], [71]. Despite sustained sexual desire, all other domains of sexual function in men were severely compromised in multiple studies [72], [73], [74]. Severe ED was present in 54% of male respondents in one French cohort [73]. In a cross-sectional case-control study of neurological disability, 100% of PD patients had abnormal sexuality, compared to 77.5% across all neurological conditions and 22.5% in controls [75].

### 4.2 Atypical parkinsonism

Sexual dysfunction is a core feature of atypical parkinsonism, with varying prevalence and severity across different syndromes.

In MSA, severe autonomic dysfunction is prominent, with ED occurring in 84–97% of men and often appearing several years before diagnosis [76], [77], [78]. Urogenital dysfunction frequently precedes motor manifestations, emphasizing its value as an early red flag autonomic symptom [76]. Female SD in MSA has been prospectively studied using the FSFI, demonstrating significantly lower scores in sexually active women with MSA compared to controls (median 15.4 vs 26.1, p = 0.0004), with the lowest scores in domains of desire, arousal, and lubrication [79]. Genital hyposensitivity was reported by 56% of women with MSA versus 9% of controls, highlighting the need for systematic assessment in women rather than extrapolating from male-centric diagnostic frameworks. [77], [79].

In PSP, urinary symptoms occur in 49–81% of patients and are associated with more rapid disease progression and shorter survival when they develop early [80], [81], [82]. Sexual problems have been reported among non-motor symptom burdens in PSP cohorts, though overall evidence remains sparse compared with MSA [80], [82], [83].

In corticobasal syndrome (CBS), limited data exist on SD specifically, though the syndrome shares some autonomic features with other atypical parkinsonian disorders [80]. Dementia with Lewy bodies (DLB) shows urinary dysfunction in approximately 42% of patients as part of the broader α-synucleinopathy spectrum [31], [80].

### 4.3 Essential tremor

Evidence on SD in essential tremor (ET) is more limited than in PD and relies primarily on non-motor symptom instruments (such as the Non-Motor Symptoms Scale (NMSS)) rather than sexual-specific questionnaires. No studies using validated sexual function measures (IIEF, FSFI) in ET populations have been identified [84], [85], [86], [87]. In ET cohorts assessed with the NMSS, patients demonstrate significantly greater non-motor symptom burden compared to healthy controls, with total NMSS scores correlating with depression, tremor severity, and age of onset [87]. While late-onset ET (≥65 years) is associated with higher rates of cognitive impairment, dementia and more rapid disease progression, the specific relationship between age and sexual domain scores in ET remains understudied, suggesting that age and broader non-motor burden may contribute to sexual dysfunction even in this primarily tremor disorder, though direct evidence is lacking [88], [89], [90].

### 4.4 Huntington's disease

In Huntington’s disease (HD), sexual disorders are also common. A systematic review found hypoactive sexual disorder in 53–83% of patients, hyperactive sexual disorder in 6–30%, ED in 48–74%, ejaculatory dysfunction in 30–65%, lubrication problems in 53–83%, and orgasmic dysfunction in 35–78% [91]. An earlier study found that 82% of HD patients and 66% of their partners had at least one sexual disorder by the Diagnostic and Statistical Manual of Mental Disorders criteria, with hypoactive sexual disorder being the most frequent [15]. In female HD patients, sexual arousal, lubrication, orgasm, and satisfaction were significantly worse than controls, though no difference in sexual desire or pain was found [92]. Severe sexual dysfunction in women correlated with Total Functional Capacity (TFC) scores below 7/13 [92].

### 4.5 Dystonia and other movement disorders

Sexual dysfunction was present in 45% of cervical dystonia patients and 39% of blepharospasm patients, versus 24% of controls [17]. In a controlled study, dystonic patients (blepharospasm and spasmodic torticollis) were more sexually compromised than patients with hemifacial spasm, with females showing poorer quality of sexual life than males [18]. A case series of 15 patients across PD, HD, Tourette syndrome and spinocerebellar ataxia type 3 documented various forms of increased sexual arousal, including hypersexuality and paraphilias [93].

### 4.6 Gender differences

Sex-related differences in sexual dysfunction in PD are likely driven by a combination of biological, sociocultural and methodological factors.

From a biological standpoint, female sexual dysfunction is influenced not only by neurodegenerative and autonomic changes related to PD but also by hormonal shifts associated with aging. These include menopause-related estrogen decline, vulvovaginal atrophy and other urogenital changes that can impair arousal, lubrication, cause pain and lead to orgasmic difficulties. In contrast, male sexual dysfunction is typically characterized by more specific outcomes, such as erectile and ejaculatory issues [1], [94], [95], [96].

A sociocultural perspective likely also influences the apparent sex disparity, as women might be less inclined to report sexual complaints spontaneously, clinicians may be less proactive in exploring them and stigma surrounding female sexuality may further reduce disclosure [8], [10], [64].

Furthermore, commonly used instruments often do not fully reflect the multidimensional aspects of female sexual dysfunction. For instance, the SCOPA-AUT includes only a few sexual items for women, potentially overlooking key areas like distress, relational context and pain. Recent research indicates that using more comprehensive, female-specific tools can lead to significantly different prevalence estimates [2], [64], [97].

Finally, women continue to be underrepresented in many PD sexual health cohorts and female dysfunction in PD is still much less studied than male dysfunction. This limits the strength of current sex-specific conclusions [9], [98].

### 4.7 Timing of onset

Sexual dysfunction can precede the motor diagnosis of PD. Pre-existing SD before PD diagnosis significantly predicted post-diagnosis SD in men [99]. ED preceded PD onset in 17.3% of cases in one study [61]. In women, reduced sexual desire increased from 36.8% before diagnosis to 52.7% after diagnosis [70]. Sexual dysfunction appeared at all PD stages [99], and notably, it did not increase with Hoehn and Yahr stage in one large questionnaire study, unlike bladder and bowel dysfunction [69].

### 4.8 Impact on quality of life and relationships

Sexual dysfunction negatively affects quality of life and partnerships. Approximately 57% of PD patients reported that the disease affected their sexual life [3]. Spousal caregivers of patients reporting inability to relax and enjoy sex and reduced libido indicated negative effects on the relationship [53]. Marital dissatisfaction was high among male patients and their partners [100]. The partner's quality of sexual life was predicted by their own younger age and better motor scores in the PD patient, while treatment with levodopa or dopamine agonists was associated with worse partner quality of sexual life [101]. In Belgium, 70% of PD patients reported dissatisfaction or avoidance related to their sex life due to the disease [59]. Yet, 60% of patients had never received any information about possible sexual health consequences of PD, and the most frequently cited barriers to discussing these issues were lack of initiative by the neurologist and embarrassment.

## 5 Associated factors

Multiple interacting factors contribute to sexual dysfunction in movement disorders, including disease-specific, medication-related, psychiatric, demographic, and hormonal variables.

### 5.1 Disease severity and motor symptoms

Significant differences in the Unified Parkinson’s Disease Rating Scale (UPDRS) scores (parts I, II, III) between patients with and without SD were found across several studies [3], [54], [60]). Disease severity as measured by Hoehn and Yahr staging correlated with SD in some cohorts [26], [102], but not in others [58], [103]. In men, motor disability level was an independent predictor of ED [104], and motor severity predicted quality of sexual life [101]. Postural instability was independently predictive of SD [55]. Sexual dysfunction was identified as an independent predictor of the postural instability and gait difficulty (PIGD) phenotype of PD [105]. In HD, sexual dysfunction progressed in parallel with functional decline (TFC) [92]. Conversely, in cervical dystonia and blepharospasm, disease duration and severity did not influence sexuality; instead, depression was the strongest predictor [17].

### 5.2 Depression and anxiety

Depression emerged as one of the most consistent predictors of sexual dysfunction across studies. Depression scores measured with the Hamilton Depression Rating Scale or the Beck Depression Inventory were significantly associated with SD in multiple cohorts [3], [8], [54], [57], [60], [63], [104]. In regression analyses, depression was the strongest predictor of loss of libido alongside age and gender [60], and the most influential factor on sexual life in men with PD [106]. Anxiety was also significantly associated with SD, particularly in females [54], [102], [107]. In women, anxiety affected body image and self-perception, contributing distinctly to SD [107]. Psychiatric factors including depression and anxiety were identified as the prevailing influences on sexual functioning in women with PD [106].

### 5.3 Demographic factors

Age was consistently identified as a predictor of SD, with older patients experiencing worse sexual functioning [8], [51], [57], [60], [69], [102]. Age was the main predictor of SD (β =  − 0.581, p = 0.006) in one Egyptian cohort [51]. Late-onset PD patients (onset > 55 years) had significantly worse ASEX scores than early-onset patients [103]. Disease duration correlated with SD in several studies [54], [74], and duration of PD was a better predictor of ED and premature ejaculation than other variables [67]. Male sex was a significant predictor in the generalized linear mixed-effects model analysis (p = 0.01) [8].

### 5.4 Medication effects

Dopaminergic therapy has a bidirectional effect on sexual function. While dopamine depletion contributes to hypoactive sexual disorders, dopamine replacement therapy, particularly dopamine agonists, can induce hypersexuality as a form of ICD [46], [108], [109], [110]. Treatment with dopamine agonists was the most important risk factor for ICD (OR = 13.39) [109]. Levodopa or dopamine agonist treatment was associated with worse partner quality of sexual life [101]. Higher doses of dopamine agonists were associated with greater risk of hypersexuality [108]. Notably, one study found that dopamine agonist use was more common among PD patients without sexual dysfunction, suggesting a possible protective or facilitative role on sexual function at standard doses [64]. Antiparkinsonian medications, including apomorphine, were implicated in psychosexual disturbances that worsened with increasing dosage [111].

Beyond dopaminergic therapies, numerous other medications commonly prescribed in movement disorders affect sexual function through diverse mechanisms. Beta-blockers, particularly propranolol used for ET, are associated with fatigue, sedation, depression and ED as major side effects, with approximately 50% of patients eventually discontinuing treatment due to limited efficacy or unacceptable side effects [112], [113]. Primidone, the other first-line agent for ET, has not been specifically associated with SD, though sedation and fatigue may indirectly affect sexual activity [88], [112]. Anticholinergic medications (trihexyphenidyl, benztropine), used for tremor and dystonia, cause peripheral antimuscarinic effects including urinary retention and dry mucous membranes that may interfere with sexual function, while their central effects (cognitive impairment, hallucinations, sedation) can indirectly affect sexual desire and performance, particularly in older adults. Katzenschlager et al., 114.

Antidepressants, frequently prescribed for depression and anxiety in movement disorders, significantly impact sexual function. SSRIs and SNRIs are associated with reduced sexual drive, delayed or absent ejaculation, difficulties achieving orgasm, impaired genital sensitivity and ED [2], [115]. A systematic review found significant associations between antidepressant therapy and decreased libido (OR = 1.89), ED (OR = 2.28) and ejaculatory dysfunction (OR = 7.31) compared to placebo [116]. Venlafaxine was associated with the highest risk of SD compared to citalopram (HR = 1.27), while mirtazapine demonstrated modestly lower risk (HR = 0.87) [117]. Importantly, some patients experience persistent sexual difficulties even after discontinuing treatment (post-SSRI sexual dysfunction), potentially due to altered serotonergic activity, peripheral axonal damage or hormonal dysregulation [2], [115].

Antiepileptic drugs used in movement disorders also affect sexual function. Enzyme-inducing antiepileptics (phenytoin, phenobarbital, carbamazepine, primidone) increase sex hormone-binding globulin concentrations, leading to reduced bioavailable testosterone and estradiol, which may cause menstrual disturbances, SD and reduced fertility [118], [119], [120]. Valproate inhibits hepatic cytochrome P450 isoenzymes and is associated with hyperandrogenism, insulin resistance, weight gain, polycystic ovarian syndrome, menstrual disorders and infertility in women [118], [119]. Topiramate and gabapentin have been associated with anorgasmia in both sexes, with topiramate-associated SD occurring even at therapeutic doses (9% frequency of self-reported SD in polytherapy) [119], [121], [122]. Enzyme-neutral antiepileptics (lamotrigine, levetiracetam, oxcarbazepine) may have more favorable sexual function profiles and may even improve sexual function in some patients [121], [123].

Vesicular monoamine transporter type 2 (VMAT2) inhibitors (tetrabenazine, deutetrabenazine, valbenazine), used for chorea in Huntington's disease and tardive dyskinesia, have not been specifically associated with SD in clinical trials, though their neuropsychiatric effects (depression, sedation, somnolence) may indirectly affect sexual function [124], [125], [126]. Benzodiazepines (clonazepam, diazepam), used for dystonia, myoclonus and tremor, may affect sexual function through sedation and potential for tolerance and dependence with long-term use [127]. Baclofen, a GABA-B receptor agonist used for spasticity and dystonia, causes sedation, drowsiness, and weakness that may indirectly impair sexual activity, though intrathecal baclofen may actually improve sexual function by reducing spasticity and improving patient comfort during sexual activities [2], [127]. Long-term opiate use for pain relief can result in low sexual desire, ED and orgasmic dysfunction through suppression of the hypothalamic–pituitary–adrenal axis, reducing free testosterone and luteinizing hormone concentrations. [2].

### 5.5 Autonomic dysfunction and hormonal factors

Autonomic dysfunction was significantly associated with SD. Patients with SD had higher total SCOPA-AUT scores and specifically higher cardiovascular, urinary and gastrointestinal autonomic subscores [26]. Urinary dysfunction was associated with SD in multivariate analysis [52], [74]. Hormonal abnormalities were documented: PD patients had significantly lower serum testosterone (450.6 vs. 534.3 ng/dL in controls) and higher cortisol [128]. Testosterone deficiency was found in 35% of male PD patients in one registry, paralleling rates in the general elderly male population [129]. In women, abnormalities in testosterone, estradiol, vitamin D3, and calcium were statistically significant [70]. These hormonal abnormalities should be interpreted in the broader context of PD and aging. Sexual function relies not just on circulating reproductive hormones but also on the regulation of the neuroendocrine axis, which aging and chronic illness can influence [130]. In PD, dopaminergic dysfunction may further disrupt endocrine balance due to dopamine's role in prolactin regulation, reproductive hormone balance, reward processing, and sexual motivation [95], [131], [132], [133], [134], [135]. Therefore, hormonal changes in PD likely result from a combination of aging, neurodegeneration and changes in dopaminergic signaling, rather than from an isolated endocrine issue.

### 5.6 Cognitive impairment

Cognitive impairment contributes to sexual dysfunction in movement disorders through bidirectional mechanisms affecting both hypoactive and hyperactive sexual behaviors. Apathy, resulting from dopamine depletion in the mesocorticolimbic system and disruption of orbitomedial/ventromedial prefrontal cortex circuits, leads to impaired emotional reactivity, reward deficiency and diminished sexual initiative [13], [136]. Executive dysfunction involving the dorsolateral prefrontal cortex and caudate nucleus impairs cognitive functions (planning, working memory, set-shifting) needed for goal-directed behavior, resulting in cognitive inertia that restricts sexual interests and activity [136]. In a controlled study of 40 men with PD assessed with the IIEF and NMSS, ED correlated inversely with age (p = 0.013) and with cognitive/mood, gastrointestinal and urinary NMSS domains, with sexual dysfunction contributing to worse quality-of-life scores, supporting the clinical relevance of cognition-related domains in sexual outcomes [51].

Conversely, cognitive impairment, particularly executive dysfunction, may paradoxically increase vulnerability to hypersexuality and ICDs by impairing inhibitory control mechanisms [137], [138], [139]. A *meta*-analysis of 34 studies demonstrated significant relationships between ICDs and dysfunction of abstraction, set-shifting, visuospatial abilities and decision-making, suggesting that PD patients with frontal dysfunctions are more vulnerable to ICDs when treated with dopaminergic medications [139]. Neuroimaging reveals that PD patients with ICDs exhibit serotonergic dysfunction within cortico-striato-pallido-thalamic circuits, with altered serotonin transporter availability and 5-HT2A receptor binding in motor and associative networks, indicating that impaired behavioral inhibition involves both dopaminergic and serotonergic systems [137].

Importantly, patients with dementia treated with dopamine agonists show higher rates of ICDs than those without dementia, indicating that cognitive impairment does not protect against but may increase susceptibility to disinhibited behaviors in the context of dopaminergic treatment [13]. This bidirectional relationship, manifesting as both hypoactive symptoms (through apathy and executive dysfunction) and hyperactive symptoms (through impaired inhibitory control), emphasizes the need for comprehensive cognitive assessment when evaluating sexual symptoms in movement disorders.

### 5.7 Interactions between factors

Importantly, these associated factors probably interact rather than function independently. The available data indicate that motor disability, autonomic dysfunction, depressive and anxiety symptoms, and other non-motor issues often occur together and collectively influence sexual dysfunction in PD. For instance, depression might partly mediate the link between increased disability and impaired sexual function, while autonomic and urinary symptoms may impact directly and indirectly by reducing comfort, self-esteem, and relationship quality. However, since most studies are cross-sectional, questions about causality and the direction of effects remain unresolved. Currently, the evidence supports a complex, multifactorial and possibly bidirectional model, rather than a straightforward one-to-one link between any single clinical variable and sexual dysfunction.

## 6 Hypersexuality

Hypersexuality merits separate discussion as a distinct clinical entity. The average lifetime prevalence of hypersexuality in PD patients on dopamine replacement therapy was 2.7% overall and 7.4% in those on dopamine agonists [108]. Prevalence rates of hypersexuality ranged from 3.5% [140] to 12.2% [109] across studies, with one cohort reporting 9.7% in young-onset PD males [67]. PD was the most frequently studied condition for hypersexuality among neurological disorders, accounting for 55.6% of studies in one systematic review encompassing 32,662 patients [141].

Hypersexuality was consistently associated with male gender, younger age, earlier PD onset, and higher dopamine agonist doses [108], [109], [142]. Onset typically occurred within 8 months of starting dopamine agonist therapy in 14 of 15 cases in one series [110]. Behaviors included compulsive masturbation, use of pornography, promiscuity, solicitation of prostitutes and paraphilias [140], [142], [143]. The impact on life was predominantly negative, affecting marital closeness, family dynamics, social interactions, work efficiency and physical health [143]. Partners of hypersexual patients frequently required psychiatric care, with four of six partners in one case series experiencing depression, suicidal intention or post-traumatic stress disorder [144].

Beyond the patient’s symptoms, hypersexuality can place significant relational and caregiver burdens. Studies on partnerships indicate that these behaviors may harm intimacy, trust and relationship stability, while partners often face distress, shame, anxiety, depression, or broader psychosocial challenges [14], [138], [143], [145]. In practical settings, screening remains difficult because embarrassment, minimization, and poor insight can lead to underreporting [21], [143]. Therefore, obtaining collateral history from the partner is especially valuable and structured tools like the Questionnaire for Impulsive-Compulsive Disorders in Parkinson’s Disease-Rating Scale (QUIP-RS) can aid systematic assessment when used alongside focused clinical interviews [97], [146].

Importantly, not all apparent hypersexuality in PD represents true compulsive sexual behavior. Careful sexological assessment revealed that among four referred cases of suspected hypersexuality, only one involved true compulsive behavior; the others reflected ED, delayed ejaculation, or desire discrepancy within the couple [147]. Additionally, hypersexuality unresponsive to dopaminergic medication reduction may signal an alternative diagnosis such as behavioral variant frontotemporal degeneration (bvFTD) overlap, as demonstrated in a case series of four patients who were reclassified from PD-ICD to bvFTD-PD overlap [148].

Neuroimaging studies identified a specific fronto-striatal and mesolimbic circuitry underlying hypersexuality, involving the caudate, pre-supplementary motor area, ventral tegmental area, and anterior cingulate cortex [149]. Dopamine medication perturbed response inhibition upon presentation of sexual cues in hypersexual patients compared to nonhypersexual peers [149].

## 7 Treatment approaches and outcomes

### 7.1 Pharmacological treatments

#### 7.1.1 Hypoactive sexual dysfunction

Pharmacological evidence for hypoactive sexual dysfunction in movement disorders is concentrated in the treatment of ED, which has been characterized as the only SD domain with evidence-based drug treatment in PD [1], [150]. In this context, phosphodiesterase-5 (PDE5) inhibitors are positioned as facilitating erectile response rather than generating erections de novo, thereby enhancing the physiological response to sexual arousal and potentially partner intimacy [150].

Randomized controlled data support sildenafil for ED in PD. In one trial, participants were randomized to 50 mg sildenafil or placebo, with dose escalation to 100 mg after 2 weeks in the absence of side effects and sildenafil significantly improved the IIEF erectile-function domain compared with placebo (p < 0.0001; mean 23.2 vs 12.3) [151]. A second randomized, double-blind, placebo controlled, crossover study in parkinsonism (12 PD and 12 MSA) used an initial 50 mg dose with adjustment for efficacy and tolerability, taken “as needed” approximately 1 h before sexual activity, and reported marked improvement in the ability to obtain and maintain an erection during active treatment. In PD, specifically, blood pressure changes were minimal between active and placebo conditions and quality of sex life improved [152]. Consistent with these trials, narrative reviews summarize sildenafil dosing as 25/50/100 mg taken orally around 1 h before intended sexual activity and cite an approximate 85% efficacy estimate in PD patients with ED and comorbid depression [150].

Beyond PDE5 inhibitors, sublingual apomorphine is presented as an additional evidence-based option for ED in PD. Reviews describe apomorphine’s pro-erectile action as mediated by a dopaminergic effect in the hypothalamus, with recommended sublingual doses of 2–4 mg and an onset of action within 10–25 min. Nausea is highlighted as the most common adverse reaction [150]. Evidence for tadalafil is sparse. In one retrospective PD cohort, only three men were reported to have received tadalafil for sexual dysfunction, without detailed outcomes [58].

Endocrine correction may be relevant in a subset of patients with hypogonadism-associated low libido and ED. Testosterone deficiency has been described as a cause of decreased libido and ED (alongside fatigue and depression) and one review reports that a daily transdermal testosterone gel improved testosterone-deficiency symptoms in men with PD, with trends toward improvement in non-motor and motor symptoms [150].

#### 7.1.2 Hyperactive sexual dysfunction

Across reviews and cohort data, first-line management emphasizes reduction or discontinuation of the dopamine agonist, despite the competing risk of worsening motor symptoms. Reviews explicitly state that hypersexuality should be treated primarily by diminishing dopamine agonists and that discontinuation is consistently effective [150]. Broader clinical guidance similarly notes that, in most cases, ICDs resolve if the dopamine agonist is withdrawn and motor symptoms are managed with levodopa monotherapy [153]. Observational data further support a temporal relationship: a case series reported that hypersexuality began within 8 months of starting dopamine agonist therapy in 14 of 15 cases and resolved in cases where the agonist was stopped despite continued levodopa treatment [110]. At the individual level, a case report described hypersexuality emerging within one month of pramipexole initiation and remitting completely after pramipexole withdrawal and substitution with ropinirole (8 mg/day) [154].

When dopamine agonist reduction is not feasible or does not fully resolve symptoms, the evidence base for adjunctive pharmacotherapy is weaker and largely derived from expert guidance and case-level experience. A review of ICD management in PD notes that the value of selective serotonin reuptake inhibitors is uncertain for ICDs triggered by dopaminergic therapies and another review reports minimal improvement in ICD behaviors with SSRIs in PD [153], [155]. The same expert review suggests that persistent ICD symptoms after dopamine agonist reduction might benefit from atypical neuroleptics such as quetiapine or clozapine [153]. Case-level experience also supports clozapine as a symptomatic control strategy for severe hypersexuality occurring after STN-DBS [156].

Outside PD, but within the broader dopaminergic-therapy context, antiandrogen therapy has been described as an adjunct when dopamine agonist cessation alone is insufficient: in a report on pramipexole-induced problematic sexual behaviors, oral medroxyprogesterone acetate (MPA) was used at 100 mg three times daily, with escalation to four times daily, with complete remission of unwanted sexual interests and behaviors after dose escalation in one case [157].

Finally, circuit-targeted noninvasive neuromodulation is emerging as a mechanistically informed approach. In PD patients with hypersexuality, excitatory repetitive transcranial magnetic stimulation (rTMS) over the presupplementary motor area improved response inhibition under sexual influence, with significant improvement for erotic (p = 0.02) and non-erotic (p = 0.04) stimuli versus sham in a theta-burst protocol [149]. Complementary experimental work using a stop-signal paradigm further supports medication-state sensitivity, a diminished response inhibition when exposed to sexual cues comparing on vs off medication, and reports improved inhibition after pre-supplementary motor area stimulation (P = 0.02 for erotic and P = 0.04 for nonerotic stimuli) [149].

### 7.2 Deep brain stimulation and sexual function

Across studies evaluating subthalamic nucleus DBS (STN-DBS) in PD, aggregated evidence suggests that sexual function outcomes are, at best, modestly improved and heterogeneous across sex and outcome measures. A systematic review of six studies reported a small statistically significant improvement in sexual function after STN-DBS, alongside a more substantial improvement in quality of life [40]. Longitudinal questionnaire data have further suggested that male, but not female, PD patients under 60 may report greater sexual satisfaction after STN-DBS [150]. In contrast, a separate *meta*-analysis focusing on erectile-function outcomes found no significant improvement in IIEF when comparing DBS ON vs DBS OFF conditions and concluded no significant association between DBS and IIEF in males [44]. Cross-sectional comparisons also underscore the continued high burden of sexual dysfunction in DBS-treated cohorts (e.g., ED present in 83.3% of men and sexual dysfunction in 77.8% of women), with age emerging as an independent predictive factor [41].

Whether DBS-related sexual improvements are direct (circuit-level) or indirect (mediated by medication reduction, motor function, mood, or relationship dynamics) is difficult to determine definitively because most available studies report net outcomes without mechanistic dissection. Some longitudinal work demonstrates that STN-DBS is followed by significant reductions in dopaminergic treatment and disease severity by Hoehn and Yahr staging, yet finds no correlation between sexual-function changes and changes in levodopa equivalent daily dose (LEDD), disease severity, depression, or anxiety. The authors explicitly conclude that their data do not allow explanation of the slight sexual-life improvement observed in men [43]. These same authors nonetheless acknowledge plausible indirect pathways, including the possibility that motor improvement may have an indirect effect on sexual functioning and that changes in marital relationship could contribute. Consistent with the general difficulty of mechanistic attribution, comparative DBS studies emphasize that they can only report “net effects” of neurostimulation plus medication reduction and they observed no linear relationship between reductions in dopaminergic therapy and outcome parameters, limiting inference about medication-mediated mechanisms [158].

Indirect pathways remain biologically and clinically plausible, even if not consistently demonstrated within individual datasets. First, STN-DBS is commonly associated with the ability to reduce dopaminergic medication burden, which could secondarily lessen dopaminergic side effects, including behavioral ICD manifestations that encompass hypersexuality [158], [159]. Large observational work in STN-DBS cohorts reported reduced prevalence of hypersexuality (12% to 8.0%, p = 0.047) over follow-up, and explicitly stated that reduction of dopaminergic medications partly explains beneficial effects on selected impulse control behaviors (ICBs), while also acknowledging that available data are insufficient to determine whether changes are due to medication decrease, modulation of oscillatory patterns, or both [160]. Second, DBS-associated motor improvement may enable sexual activity by reducing disability, a facilitation mechanism rather than a specific sexual-circuit effect [156]. Third, relationship and caregiver dynamics are repeatedly raised as potential mediators: one STN-DBS study explicitly states that changes in the marital relationship could also be involved in sexual satisfaction changes after surgery [43]. Finally, DBS can influence psychological well-being through psychosocial and coping factors alongside medication modifications and stimulation effects, providing a conceptual basis for mood/adjustment-mediated sexual outcomes even when direct correlations are absent in specific cohorts [161].

At the same time, several lines of evidence suggest that direct circuit-level effects, or stimulation-location–dependent effects on sexual behavior are plausible, particularly for hypersexuality and reward-seeking phenotypes. Case reports describe de novo hypersexuality after STN-DBS occurring despite marked motor improvement and >50% drug reduction, with authors arguing that medication effects or electrode misplacement are unlikely explanations in those cases and proposing a direct dopaminergic/circuit mechanism: excess dopamine made available following STN DBS could result in hypersexuality, and/or variable stimulation of motor versus limbic STN divisions or current spread to surrounding structures [156]. Beyond single cases, mechanistic reviews of dopaminergic dysregulation syndromes after STN-DBS propose that appearance or worsening of behavioral addictions could relate to stimulation or misplacement within the medial STN limbic territory, and describe neuroimaging evidence of STN-DBS–induced metabolic changes in cortical and subcortical regions related to limbic and associative circuits. In de novo cases, the combined effect of even reduced dopaminergic therapy plus DBS in limbic STN territory is hypothesized to hyperstimulate the mesolimbic system [162]. More generally, direct induction of neuropsychiatric syndromes by STN-DBS including hypomanic/manic states is well documented and reversible by stimulation-parameter changes, supporting the principle that stimulation of non-motor STN subareas can exert direct limbic and reward-related effects that could, in some patients, extend to sexual drive and disinhibition [161].

Table 2 summarizes the impact of commonly used medications in the field of movement disorders on sexual function.

**Table 2:** Medications commonly used in movement disorders and their impact on sexual function. COMT: catechol-o-methyltransferase; DBS: deep brain stimulation; ED: erectile dysfunction; ICD: impulse control disorder; MAO-B: monoamine oxidase-B; PCOS: polycystic ovary syndrome; PD: Parkinson’s disease; PDE5: phosphodiesterase-5; REM: rapid eye movement; SD: sexual dysfunction; SNRI: serotonin and norepinephrine reuptake inhibitor; SSRI: selective serotonin reuptake inhibitor; VMAT2: vesicular monoamine transporter type 2.

Medication	Impact on Sexual Health	Recommendations
Levodopa [11], [38], [119], [163]	Can improve sexual function by improving motor symptoms. May cause hypersexuality (less common than with dopamine agonists). ED can occur but less frequently than with dopamine agonists.	Dose adjustment if hypersexuality occurs. Consider adding PDE5 inhibitor for ED if no contraindications.
Dopamine agonists [11], [20], [38], [119], [163], [164]	High risk of hypersexuality and ICD (4timeshigherthanlevodopa). Can also improve ED and libido in some patients.	Screen regularly for ICD. Reduce dose or discontinue if hypersexuality develops. Consider switching to levodopa. For ED, may benefit from the medication itself or add PDE5 inhibitor.
MAO-B inhibitors [165], [166]	Generally minimal direct sexual side effects. Other side effects (insomnia, cognitive impairment) can indirectly affect sexual function.	If SD occurs, evaluate for other contributing medications or disease-related factors. Avoid combining with SSRIs/SNRIs due to serotonin syndrome risk.
COMT inhibitors [165]	Minimal direct sexual side effects.	If SD occurs, evaluate for other contributing medications or disease-related factors.
Amantadine [165]	Minimal direct sexual side effects. Anticholinergic-like effects (dryness) can indirectly affect sexual function.	If SD occurs, evaluate for other contributing medications. Reduce dose if cognitive or anticholinergic side effects are problematic.
Anticholinergics [166]	May indirectly impair sexual function through cognitive effects, urinary symptoms or dryness.	Reduce dose or discontinue if urinary retention or cognitive impairment occurs.
Propranolol [88], [112], [113]	ED is a common side effect. Fatigue and depression may indirectly impair sexual function.	Switch to primidone or other tremor medications. Add PDE5 inhibitors for ED. Reduce dose if possible.
Primidone [88], [112], [113]	Minimal direct sexual side effects. Sedation, nausea, confusion may occur at initiation and impair indirectly sexual function.	Start at low dose and titrate slowly. If SD occurs, evaluate for other contributing factors.
Topiramate [88], [118], [119], [127]	Reversible anorgasmia reported in case reports. Paresthesia and cognitive impairment may indirectly impair sexual function.	Discontinue or reduce dose if anorgasmia occurs. Switch to alternative tremor or myoclonus or tic medication.
VMAT2 inhibitors [124]	Minimal direct sexual side effects. Sedation and depression may indirectly impair sexual function.	Monitor closely for depression. Consider dose reduction or switching to valbenazine for better tolerability.
Valproate [118], [119], [127]	In women: hyperandrogenism, PCOS, menstrual disorders, infertility, weight gain. In men: increased testosterone, weight gain. Mood disturbances.	Avoid in women of childbearing potential. Switch to alternative antimyoclonic agents.
Levetiracetam [119], [127]	Minimal direct sexual side effects.	Preferred antimyoclonic agent when sexual function is a concern.
Gabapentin [112], [119]	Reversible anorgasmia and ED reported. Sedation and dizziness may indirectly affect sexual function.	Discontinue or reduce dose. Switch to alternative tremor medication.
Carbamazepine [118], [119]	Decreased libido, ED.	Switch to enzyme-neutral antiepileptic (levetiracetam). Add PDE5 inhibitor for ED. Monitor hormone levels.
Baclofen [167]	ED, decreased libido. Sedation, weakness and cognitive impairment may indirectly affect sexual function.	Reduce dose if possible. Consider intrathecal baclofen. Add PDE5 inhibitors for ED. Switch to tizanidine or botulinum toxin.
SSRIs, SNRIs, tricyclic antidepressants [2], [116], [168], [169]	High rates of SD (25–80%): decreased libido, delayed/absent orgasm, ED, impaired genital sensitivity. Higher risk of ED with SNRIs. Possible anticholinergic effects worsening sexual function with tricyclic antidepressants.	Switch to antidepressants with lower sexual side effects (bupropion, mirtazapine, nefazodone, vilazodone). Add PDE5 inhibitor for ED.
Trazodone [170], [171], [172]	Can cause priapism. Lower SD rates than SSRIs.	Useful for insomnia in PD. Monitor for priapism. Avoid or use cautiously if REM sleep behavior disorder present.
Antipsychotics [173], [174], [175]	Very high rates of SD (up to 60%): decreased libido, ED, ejaculatory dysfunction.	Generally avoided in PD due to motor worsening. If used, switch to quetiapine or clozapine or pimavanserin. Aripiprazole has the lowest SD rates among antipsychotics (16–20%).
Benzodiazepines [176]	Changes in libido, ED, decreased orgasm. Sedation and cognitive effects may indirectly impair sexual function.	Reduce dose or discontinue if possible. Use lowest effective dose. Avoid in elderly with cognitive impairment.
Melatonin [163], [166], [177], [170], [171], [172]	No significant sexual side effects.	Preferred for insomnia and REM sleep behavior disorder in PD. No dose adjustment needed for SD.
Modafinil [170], [172]	Minimal sexual side effects. May improve daytime alertness which could indirectly benefit sexual function.	Useful for excessive daytime sleepiness in PD. No dose adjustment needed for SD.
Botulinum toxin [2], [170]	No systemic sexual side effects. Experimental use in cavernosal tissue for ED.	No dose adjustment needed for SD.
DBS [2], [40], [41], [119], [178]	May improve sexual function through improvement in motor symptoms and quality of life. Hypersexuality reported in some cases.	Monitor for hypersexuality. Adjust stimulation parameters if hypersexuality occurs.

### 7.3 Non-pharmacological approaches

Non-pharmacological management includes multidisciplinary counseling, occupational therapy for physical adaptations, management of secondary symptoms and emerging neuromodulation techniques. These interventions address the multidimensional nature of SD, targeting physical, psychological and relational factors.

Psychosexual counseling helps address perceived changes in body image and self-esteem that commonly affect intimacy in patients with movement disorders [2]. Relationship counseling can address altered dynamics between the patient and partner, particularly when the partner assumes caregiving responsibilities that may affect the sexual relationship [2], [3], [179]. The PLISSIT model (Permission, Limited Information, Specific Suggestions and Intensive Therapy) provides a structured framework for initiating and managing these sensitive conversations in the clinical setting [2], [180]. Mental health support is appropriate when depression and anxiety contribute to sexual performance difficulties [2], [8], [106]. Mindfulness-based interventions and yoga have shown beneficial effects on anxiety and depressive symptoms in PD, with secondary improvements in quality of life, which may indirectly benefit sexual function [2], [181].

Occupational therapists can provide practical support for addressing physical disabilities that affect sexual activity, including specific suggestions for positioning when spasticity and poor mobility create barriers to intimacy. Physical therapy interventions may include mobility training to optimize bed mobility for sexual positioning and transferring, as well as management of spasticity using techniques such as positioning aids [2], [182].

Targeting secondary symptoms can significantly improve sexual function. Treatment of lower urinary tract and bowel dysfunction reduces fear of incontinence during sexual activity, with studies showing that incontinence treatment is associated with improved sexual function. Managing pain, spasticity and fatigue, common symptoms that negatively affect intimacy and sexual desire, forms an important component of comprehensive care [2].

Transcutaneous tibial nerve stimulation, used for neurogenic overactive bladder, has shown improvement in sexual function in both men and women. Other investigational approaches include clitoral nerve stimulation and transcutaneous electrical stimulation of the dorsal penile nerve, though these require validation in larger studies [2], [183], [184].

A comprehensive strategy requires addressing multiple domains simultaneously. Most patients with PD express interest in receiving sexual counseling, with over 70% of men and 40% of women motivated to discuss these issues [99]. The holistic management of SD in movement disorders necessitates coordination among neurologists, mental health professionals, occupational therapists and other specialists to address the complex interplay of neurogenic, physical, psychological and relational factors [1], [2].

**Fig. 2:**
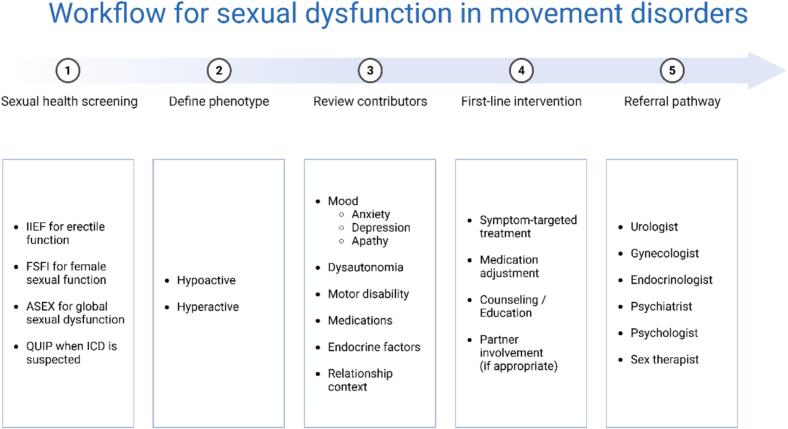
Suggested clinical workflow for sexual dysfunction in movement disorders. ASEX: Arizona Sexual Experience Scale; FSFI: Female Sexual Function Index; ICD: Impulse Control Disorder; IIEF: International Index of Erectile Function; QUIP: Questionnaire for Impulsive-Compulsive Disorders in Parkinson's Disease.

In routine practice, sexual health may be more effectively addressed when it is embedded into standard movement disorder care rather than deferred to patient self-report. A pragmatic first step is to normalize the topic during consultation, use brief screening questions or validated tools when appropriate and distinguish hypoactive symptoms from hypersexuality. Patients with persistent symptoms, marked distress, complex couple-related difficulties, endocrine concerns, genitourinary symptoms or suspected compulsive sexual behavior may then be referred to the most relevant specialist, including urology, gynecology, endocrinology, psychiatry, psychology or sex therapy. A structured multidisciplinary pathway may help clinicians move from under-recognition to actionable management in everyday practice. A practical summary of this workflow, including potential first-line tools and referral pathways, is provided in Fig. 2.

## 8 Future directions

SD is consistently described as one of the most neglected non-motor symptoms in movement disorders, particularly PD. A 2025 review in Movement Disorders Clinical Practice frames this as an urgent unmet need in neurological clinics and a 2025 report from the Fifth International Consultation on Sexual Medicine reinforces the same conclusion: sexual health should be a core component of neurological rehabilitation, yet currently it rarely is. Several directions are emerging as priorities [185], [186].

Across the entire field of neurogenic SD, the only intervention with robust evidence is PDE5 inhibitors for ED, a pattern that has remained essentially unchanged for years. Filling this gap with well-designed RCTs, especially for women, and for orgasmic, desire and arousal domains beyond erectile function, is a research priority [187].

In PD specifically, dopamine agonist-induced hypersexuality represents a clinically important problem, with real social, psychological and legal consequences [21]. Pathophysiologically, the mechanisms linking mesocortical dopamine dysregulation to reward-circuit hypersensitivity remain incompletely understood and there are no pharmacological agents with established evidence for treating this complication beyond reducing or discontinuing the offending drug. Developing better predictive biomarkers and targeted treatments for ICD in this context is a clear gap.

Several non-pharmacological interventions: pelvic floor muscle training, mindfulness-based programs, structured sexual counseling, exercise and assistive devices, show promising signals [188]. These remain understudied in movement disorder populations and rigorous trials are needed. A 2025 clinical roadmap for PD specifically called for sexual health guidelines covering women with PD, noting that the evidence base has historically been generated almost exclusively in men [189].

The effect of SD on partners and relationship quality is recognized as a key unmet need, yet few studies formally include partners as subjects or measure couple-level outcomes [1]. This is a methodological frontier that would align research design more closely with what patients themselves identify as meaningful.

A recurring finding is that both patients and clinicians avoid initiating discussions about sexual health and that this avoidance is itself a major barrier to care [99], [190]. Structured interventions, including validated screening tools embedded in clinical workflows and training programs for movement disorder specialists, could be proposed as feasible near-term steps. One prospective study found that more than 70% of PD patients were motivated to receive sexual counseling when directly asked, suggesting demand far exceeds current supply.

The overall picture is a field that has accurately diagnosed its own gaps for some time, but where high-quality interventional evidence, especially for women, for non-erectile outcomes and for conditions other than PD, remains genuinely sparse.

### 8.1 Synthesis

The literature consistently demonstrates that sexual dysfunction is a highly prevalent non-motor feature of PD and other movement disorders, yet the wide range of reported prevalence (23–95%) and the variable significance of individual risk factors demand careful interpretation.

The most apparent source of heterogeneity in prevalence estimates is measurement methodology. Studies using the ASEX with a threshold of ≥19 report lower rates (23%) [59], while those using comprehensive, domain-specific instruments such as the IIEF/FSFI or broad clinical criteria report rates exceeding 80% [55], [56]. The single study found no overall difference between PD patients and controls enrolled exclusively mild-to-moderate patients and used four parallel scales, suggesting that in early-stage PD with preserved function, sexual impairment may not yet differentiate from age-matched norms [65]. Studies with higher prevalence typically included patients across all Hoehn and Yahr stages or used lower thresholds for dysfunction.

The gender discrepancy, with *meta*-analytic evidence for significant SD association in males but not females [19], likely reflects both physiological and methodological factors. ED is a discrete, easily assessed endpoint, whereas female sexual dysfunction involves more diffuse domains (desire, arousal, lubrication, orgasm, pain) that are harder to capture and may be confounded by menopausal changes. Additionally, women with PD may underreport SD due to cultural factors, and fewer studies have specifically investigated female populations [64], [70], [98].

Among associated factors, depression emerges as the single most robust and potentially modifiable predictor of SD across virtually all populations studied, male and female, early and late onset, and across disease severities [8], [60], [63], [106]. Critically, loss of libido resulting from depression was not associated with antidepressant use [60], suggesting that depression-related SD may be undertreated. Age is the second most consistent predictor [8], [51], [60], and its effect is difficult to disentangle from age-related hormonal decline, comorbidities, and longer disease exposure. In addition, dopaminergic and neuroendocrine changes may interact with aging-related hormonal shifts, further complicating interpretation of hormonal correlates in PD. Disease severity (UPDRS, Hoehn & Yahr stage) shows a more variable relationship, significant in some studies [26], [102] but not others [58], [103], possibly because SD can emerge early in the disease before substantial motor impairment, and because the relationship may be non-linear, with autonomic and dopaminergic dysfunction contributing independently of motor staging. These observations support the view that sexual dysfunction in PD is not explained by a single determinant, but more likely reflects the cumulative and interacting effects of mood symptoms, autonomic dysfunction, disability, aging and treatment exposure.

The bidirectional role of dopaminergic medication is clinically important. At physiological replacement levels, dopaminergic therapy may preserve or improve sexual function, as evidenced by improvements with pergolide [191], [192] and the observation that dopamine agonist users were less likely to have SD [64]. At supratherapeutic levels, particularly with dopamine agonists, hypersexuality emerges, mediated through mesolimbic reward circuitry involving the caudate – pre-supplementary motor area – ventral tegmental area – anterior cingulate cortex network [149]. The prevalence of hypersexuality increases from approximately 2.7% overall to 7.4% in patients on dopamine agonists [108], and the strongest single pharmacological risk factor for ICD is dopamine agonist use (OR = 13.39) [109]. Resolution upon agonist withdrawal [110], [140] confirms the causal role, though cases unresponsive to medication reduction may indicate alternative pathology such as FTD overlap [148].

Treatment evidence remains sparse and methodologically limited. The strongest data support PDE5 inhibitors for ED (84.8% improvement with sildenafil) [193], dopamine agonist withdrawal for hypersexuality [110], and STN-DBS for modest improvements in sexual satisfaction in younger males (standardized mean difference =  − 0.124) [40]. The rTMS evidence for hypersexuality is novel but preliminary, drawn from a single sham-controlled study of 20 patients [149], [149]. No randomized controlled trials specifically targeting sexual dysfunction in PD were identified. The absence of treatment studies in women is particularly striking given the high prevalence of SD in this population.

In dystonia, the pattern of SD differs mechanistically: disease severity and duration do not predict SD, while depression does [17], suggesting that SD in dystonia may be more psychologically mediated, possibly through altered body image and self-consciousness about physical appearance, rather than reflecting the same autonomic and dopaminergic deficits operative in PD [18]. In HD, the coexistence of hypoactive and hyperactive sexual disorders within the same population [15], [91] highlights the complex interplay of cognitive decline, behavioral disinhibition, and neurodegeneration affecting both cortical and subcortical circuits.

Across all movement disorders, sexual dysfunction remains systematically under-recognized and under-treated. Sixty percent of PD patients had never received information about sexual health consequences [59], and men were more likely than women to receive treatment for sexual symptoms despite comparable symptom burdens [194]. These findings underscore the need for routine screening, ideally from the time of diagnosis [59], using validated instruments, combined with multidisciplinary management addressing pharmacological, psychological, hormonal, and relational dimensions of sexual health in this population.

## 9 Declaration of generative AI and AI-assisted technologies in the manuscript preparation process

No AI and AI-assisted technologies were used during the preparation of this work.

## CRediT authorship contribution statement

**Vincent Leclercq:** Writing – original draft, Visualization, Project administration, Methodology, Investigation, Data curation, Conceptualization.

## Funding

This research received no specific grant from any funding agency in the public, commercial, or non-profit sectors.

## Declaration of competing interest

The author declares that he have no known competing financial interests or personal relationships that could have appeared to influence the work reported in this paper.
